# Lycopene and SKQ1 Improve Boar Sperm Quality During 17 °C Storage via the AMPK/Nrf2 Pathway

**DOI:** 10.3390/antiox14121391

**Published:** 2025-11-21

**Authors:** Miaolian Peng, Pengyao Wang, Yongchang Lu, Xiaoliang Wang, Xianwei Zhang, Ruhai Xu, Ting Gu, Gengyuan Cai, Zhenfang Wu, Lihe Dai, Linjun Hong

**Affiliations:** 1 Guangdong Laboratory for Lingnan Modern Agriculture, State Key Laboratory of Swine and Poultry Breeding Industry, National Engineering Research Center for Breeding Swine Industry, Guangdong Provincial Key Laboratory of Agro-Animal Genomics and Molecular Breeding, College of Animal Science, South China Agricultural University, Guangzhou 510642, China; kepeng0421@163.com (M.P.); pengyao@stu.scau.edu.cn (P.W.); yongchang_lu@stu.scau.edu.cn (Y.L.); wangxiaoliang2024@stu.scau.edu.cn (X.W.); tinggu@scau.edu.cn (T.G.); cgy0415@scau.edu.cn (G.C.); wzf@scau.edu.cn (Z.W.); 2 Yunfu Subcenter of Guangdong Laboratory for Lingnan Modern Agriculture, Yunfu 527300, China; zxianw@163.com; 3 Zhejiang Key Laboratory of Livestock and Poultry Biotech Breeding, Institute of Animal Husbandry and Veterinary Sciences, Zhejiang Academy of Agricultural Sciences, Hangzhou 310021, China; xurh@zaas.ac.cn; 4 National Regional Gene Bank of Livestock and Poultry (Gene Bank of Guangdong Livestock and Poultry), Guangzhou 510642, China

**Keywords:** boar sperm, lycopene, SKQ1, 17 °C storage, metabolomics

## Abstract

During the storage of boar sperm at 17 °C, reactive oxygen species (ROS) are continuously generated. Excessive ROS can disrupt the mitochondrial redox balance and cause sperm damage. In this study, boar semen was diluted with solutions containing different concentrations of the natural antioxidant lycopene (1, 5, 10, 20, 40, 80 μM) or the mitochondria-targeted antioxidant SKQ1 (1, 5, 10, 25, 50, 70 nM), and sperm vitality was assessed throughout storage at 17 °C. Based on the screening results, the optimal concentrations were selected for combined application to investigate their effects on sperm quality and potential synergistic interactions. The results demonstrated that sperm motility was significantly higher in the 20 μM lycopene and 50 nM SKQ1 treatment groups compared to the control (*p* < 0.05). The combined treatment of 20 μM lycopene and 25 nM SKQ1 exhibited a synergistic effect, significantly improving sperm vitality, acrosome and membrane integrity, superoxide dismutase (SOD), glutathione peroxidase (GSP), adenosine triphosphate (ATP) levels (*p* < 0.05). Meanwhile, ROS and malondialdehyde (MDA) levels were significantly reduced (*p* < 0.05). Metabolomics analysis identified 52 differential metabolites (*p* < 0.05), including ABC transporters, corticosterone, and palmitic acid. KEGG pathway enrichment analysis revealed that these metabolites were mainly associated with steroid hormone biosynthesis, ABC transporters, and AMPK signaling pathways (*p* < 0.05), most of which were related to sperm cell energy metabolism and signal transduction. Furthermore, treatment with antioxidants significantly increased p-AMPK and Nrf2 expression in sperm cells (*p* < 0.05). These findings suggest that the combination of lycopene and SKQ1 improves boar sperm quality during 17 °C storage by enhancing energy metabolism and mitigating oxidative stress, potentially through the activation of the AMPK/Nrf2 pathway.

## 1. Introduction

With the widespread use of artificial insemination technology in the livestock industry, semen preservation techniques have developed rapidly [1]. Currently, boar semen preservation is mainly divided into two methods: cryopreservation and 17 °C liquid preservation [2]. However, after freezing and thawing, the fertility rates of artificial insemination are significantly lower compared to those using fresh semen. In fact, only 1% of all artificial inseminations worldwide involve the use of cryopreserved boar semen [3]. In practice, the 17 °C liquid preservation technique is more commonly used. This method effectively suppresses sperm motility and reduces energy consumption, allowing sperm to be stored for longer periods ex vivo. However, during storage, sperm mitochondria continuously produce ROS [4]. Boar sperm contain a high amount of polyunsaturated fatty acids [5] and a low cholesterol/phospholipid ratio [6], making them more vulnerable to ROS-induced damage. This leads to lipid peroxidation, oxidative stress, and mitochondrial dysfunction, which impair sperm vitality. Studies have shown that the addition of antioxidants in semen diluents can effectively alleviate oxidative damage to boar sperm [7,8], thereby improving preservation outcomes.

Lycopene is a natural carotenoid primarily found in mature tomatoes, watermelon, and other fruits and vegetables [9]. It contains 11 conjugated double bonds and is considered the most potent antioxidant in the carotenoid family, with antioxidant activity twice that of β-carotene and ten times that of α-tocopherol [10]. Increasing research emphasizes its significant role in treating reproductive dysfunctions [11]. Studies by Eva Tvrdá [12], Sheikholeslam [13], and Bintara Sigit [14] have shown that lycopene can scavenge ROS and, as an antioxidant, effectively prevent sperm damage caused by oxidative stress, thereby improving sperm quality in cattle, dogs, and sheep.

SKQ1 is a mitochondrial-targeted antioxidant that can actively penetrate the cell membrane and accumulate within the mitochondria. It has high membrane penetration capabilities and a strong antioxidant ability to eliminate ROS [15,16], with remarkable efficacy even at low concentrations in the nanomolar range [17]. Li et al. [18] demonstrated that supplementing SKQ1 during in vitro fertilization could mitigate oxidative stress, enhancing the maturation rate of mouse oocytes and the subsequent development of in vitro fertilized embryos. Jiang et al. [19] found that SKQ1 effectively improved spermatogenesis in Immp2l mutant mice and may be used in the treatment of male infertility. Currently, mitochondrial-targeted antioxidants such as Mito-Tempo [20] and MitoQ [21] have been studied in the context of sperm preservation, demonstrating protective effects that enhance sperm quality. However, there is limited research on the impact of SKQ1 on sperm.

To date, no studies have assessed the combined use of lycopene and SKQ1 in boar semen stored at 17 °C. Therefore, the aim of this study is to assess the protective effects of these two antioxidants on boar sperm, identify the optimal treatment concentrations, and explore the combined effects of these antioxidants to maximize sperm quality during storage.

## 2. Materials and Methods

### 2.1. Chemical Reagents

Routine chemicals and reagents were purchased from Sigma-Aldrich Agricultural Technology Development Co., Ltd. (Shanghai, China), Beyotime Biotechnology Institute (Shanghai, China), and Nanjing Jiancheng Bioengineering Institute (Nanjing, China). lycopene and SKQ1 were purchased from MedChemExpress Biotechnology Co., Ltd. (Shanghai, China).

### 2.2. Preparation of Basic Dilution Medium and Semen Collection

The basic dilution medium was prepared with the following composition: 2000 mg of glucose, 1200 mg of fructose, 675 mg of Tris, 500 mg of Hepes, 500 mg of sodium citrate, 250 mg of potassium chloride, 210 mg of citric acid, 100 mg of sodium bicarbonate, 200 mg of EDTA, 100 mg of penicillin, and 100 mg of streptomycin, all dissolved completely in 100 mL of ultrapure water. The semen samples used in this study were collected from 10 sexually mature and healthy Yorkshire boars reared by the Wens Group (Yunfu, China). Semen was collected using an artificial vagina, and the gel portion was removed using a double-layer sterile gauze. Semen samples from individual boars were kept separate throughout the experiment and were treated as independent biological replicates to account for inter-boar variability. After collection, the semen was transferred to a vacuum cup and bring to the laboratory within 30 min to dilute the density to 1.2 billion per 50 mL.

### 2.3. Semen Analysis and Evaluation

Sperm motility and morphology were assessed using a Computer-Assisted Sperm Analysis (CASA) system (Barcelona, Spain). A quantity of 5 μL of the semen was placed into a pre-warmed eight-chamber counting slide designed for boar sperm, and observed under an optical microscope at 40 × magnification [22]. Five random fields were selected for analysis, with at least 500 sperm cells evaluated. The sperm used in the experiment were required to meet the following criteria: motility ≥ 85% and morphological abnormality rate ≤ 10%.

### 2.4. Semen Treatment

After evaluating the quality of fresh boar semen samples, the samples were grouped, with at least three repetitions per group. The control group was supplemented with an equal volume of semen extender, while the experimental groups were supplemented with lycopene (1, 5, 10, 20, 40, 80 μM) and SKQ1 (1, 5, 10, 25, 50, 70 nM). The samples were then placed in a 17 °C incubator, with inversion every 8 h to prevent sperm sedimentation and aggregation.

### 2.5. Sperm Acrosome Integrity Assessment

Semen samples were centrifuged at 1500× *g* for 4 min at room temperature to remove the supernatant, and the pellets were resuspended in 1 mL DPBS. Subsequently, 20 μL of semen was placed on a glass slide and air-dried in a laminar flow cabinet. Then, 30 μL of anhydrous methanol was added for fixation at room temperature for 10 min, followed by three washes with TBST. Acrosomal integrity was assessed by FITC-PNA (L-7381, Sigma-Aldrich, Shanghai, China) staining, which allows visualization of the acrosomal structure, and Hoechst 33,342 (HY-15627, MedChemExpress, Shanghai, China) staining to observe the sperm nucleus, determining whether the acrosomal structure is intact. Afterward, 100 μg/mL of peanut agglutinin (FITC-PNA) and 10 μM of Hoechst 33342 were added to cover the semen completely. The samples were incubated at room temperature in the dark for 30 min, followed by three washes with TBST. Finally, images were captured under a fluorescence microscope, and at least 200 sperm cells were counted per group.

### 2.6. Sperm Membrane Integrity Assessment

Semen samples were centrifuged at 1500× *g* for 4 min at room temperature to remove the supernatant, and the pellets were resuspended in 1 mL DPBS. The sperm membrane integrity was assessed using 6-CFDA and PI staining. 6-CFDA stains intact sperm green, while PI enters damaged sperm membranes and stains the sperm head red. To assess membrane integrity, 10 μM 6-CFDA (HY-D0721, MedChemExpress, Shanghai, China) and 10 μM PI (HY-D0815, MedChemExpress, Shanghai, China) were added to a 1.5 mL centrifuge tube and incubated in a 37 °C water bath for 10 min. Then, 20 μL of semen was placed on a glass slide, covered with a coverslip. Finally, images were captured under a fluorescence microscope, and at least 200 sperm cells were counted per group.

### 2.7. Sperm ROS Content Measurement

The Reactive Oxygen Species (ROS) content was measured using a ROS detection kit (Beyotime Biotechnology, Shanghai, China). One milliliter of sperm sample was mixed with 200 μL of DCFH-DA working solution and incubated at 37 °C in the dark for 15 min. Fluorescence intensity was then measured using a multifunctional microplate reader (Synergy H1 Hybrid, Bioteke, Winooski, VT, USA).

### 2.8. Sperm GSP Content Measurement

Glutathione peroxidase (GSP) activity was assessed with a commercial assay kit (Nanjing Jiancheng Bioengineering Institute, Nanjing, China). Semen samples were centrifuged (1500× *g*, 4 min), and the resulting pellets were resuspended in PBS. Following thorough homogenization and lysis, the lysates were centrifuged at 12,000× *g* for 5 min to collect the supernatant. The assay working solution was then added to the supernatant as per the kit instructions, and the absorbance was immediately measured using a Synergy H1 Hybrid multifunctional microplate reader (Bioteke, Winooski, VT, USA).

### 2.9. Sperm SOD Activity Measurement

The total superoxide dismutase (SOD) activity was measured using the WST-8 method (Beyotime Biotechnology, Shanghai, China). Following the kit’s instructions, semen samples were lysed thoroughly, and then centrifuged at 12,000× *g* for 5 min. The WST-8/enzyme working solution was added, and the mixture was incubated at 37 °C for 30 min. The absorbance was measured using a multifunctional microplate reader (Synergy H1 Hybrid, Bioteke, Winooski, VT, USA).

### 2.10. Sperm MDA Content Measurement

Malondialdehyde (MDA) content was assessed with a commercial lipid peroxidation assay kit (Beyotime Biotechnology, Shanghai, China). Semen samples were centrifuged (1500× *g*, 4 min), and the pellets were resuspended in PBS. The sperm suspension was then mixed with the MDA detection working solution and heated in a boiling water bath for 15 min. After cooling to room temperature, the mixture was centrifuged at 1000× *g* for 10 min. The resulting supernatant was collected, and its absorbance was measured using a Synergy H1 Hybrid multifunctional microplate reader (Bioteke, Winooski, VT, USA).

### 2.11. Sperm MMP Level Measurement

Mitochondrial membrane potential (MMP) was measured using a MMP detection kit (Beyotime Biotechnology, Shanghai, China). According to the manufacturer’s instructions, semen samples were centrifuged at 1500× *g* for 4 min at room temperature to remove the supernatant, and the pellets were resuspended in PBS. The samples were then incubated with JC-1 working solution. When mitochondrial membrane potential is high, JC-1 aggregates in the mitochondrial matrix, forming polymers that emit red fluorescence. When the membrane potential is low, JC-1 remains in monomeric form and produces green fluorescence. The fluorescence intensity was measured using a flow cytometer (CytoFLEX, Beckman Coulter, Shanghai, China).

### 2.12. Sperm ATP Level Measurement

ATP concentration was quantified using a dedicated assay kit (Beyotime Biotechnology, Shanghai, China). Semen samples were processed according to the kit instructions. Briefly, samples were centrifuged at 1500 × *g* for 4 min at room temperature. The pellet was resuspended in DPBS, and 100 μL of this suspension was added to a 96-well plate. An equal volume (100 μL) of the ATP detection working solution was then added to each well. After homogenization by shaking for 2 min, the plate was incubated at room temperature for 10 min. The luminescent signal was recorded immediately using a Synergy H1 hybrid multi-mode microplate reader (Bioteke, Winooski, VT, USA).

### 2.13. Western Blotting Analysis

Semen samples were centrifuged at 1500 × *g* for 4 min at room temperature. After discarding the supernatant, the pellets were lysed in RIPA buffer supplemented with 1% protease inhibitor. Protein concentrations were determined using a BCA Protein Assay Kit (Beyotime Biotechnology, Shanghai, China) in accordance with the manufacturer’s protocol. Proteins were resolved electrophoretically on 10% SDS-polyacrylamide gels and subsequently transferred onto PVDF membranes. The membranes were then blocked with 2% BSA for 2 h at room temperature. Following blocking, three washes were performed with 1× TBS. The membranes were incubated overnight at 4 °C with specific primary antibodies (refer to Table 1), followed by another three washes with 1× TBST. Thereafter, the membranes were treated with corresponding secondary antibodies. Protein signals were visualized using an enhanced chemiluminescence (ECL) detection reagent (Beyotime, Cat. No. P0018S, Shanghai, China) and imaged with a UVP analysis system (Upland, Upland, CA, USA) [23]. Following image acquisition, the band intensity (optical density) of both the target proteins and the β-actin loading control was quantified using ImageJ software, version 1.54p (National Institutes of Health, Bethesda, MD, USA). For each sample, the relative expression level of the target protein was calculated by normalizing the optical density value of the target protein band to that of the corresponding β-actin band. Each Western blot experiment was independently repeated at least three times.

### 2.14. Mass Spectrometry Analysis

#### 2.14.1. Metabolite Extraction

Weigh an appropriate amount of the sample and transfer it into a 2 mL centrifuge tube. Add 1000 µL of tissue extract (75% methanol:chloroform, 9:1; 25% H_2_O) along with steel balls. Place the tube in a tissue grinder and grind at 50 Hz for 60 s, repeating the process twice. Perform ultrasonic treatment at room temperature for 30 min, followed by an ice bath for 30 min. Next, centrifuge the mixture at 12,000 rpm for 10 min at 4 °C. Collect all the supernatant, transfer it to a new 2 mL centrifuge tube, and concentrate and dry the sample. Finally, add 200 µL of a 50% acetonitrile solution prepared with 2-chloro-l-phenylalanine (4 ppm) to re-dissolve the sample. Filter the supernatant through a 0.22 μm membrane and transfer it to a detection bottle for LC-MS analysis [24].

#### 2.14.2. Liquid Chromatography Conditions

Liquid chromatography analysis was conducted on a Vanquish UHPLC system (Thermo Fisher Scientific, Waltham, MA, USA) equipped with an ACQUITY UPLC ^®^ HSS T3 column (2.1 × 100 mm, 1.8 µm; Waters, Milford, MA, USA), which was maintained at 40 °C. The mobile phase flowed at 0.3 mL/min, and a 2 µL aliquot was injected for analysis. For positive ionization mode [ESI(+)], the mobile phase consisted of 0.1% formic acid in water (A2) and 0.1% formic acid in acetonitrile (B2). The analytes were eluted using the following gradient: 8% B2 (0–1 min), increased to 98% B2 (1–8 min), held at 98% B2 (8–10 min), returned to 8% B2 (10–10.1 min), and re-equilibrated at 8% B2 (10.1–12 min). For negative ionization mode [ESI(-)], the separation utilized 5 mM ammonium formate in water (A3) and pure acetonitrile (B3), with an identical gradient profile as described for the ESI(+) mode [25].

#### 2.14.3. Mass Spectrum Conditions

Metabolite detection was carried out on an Orbitrap Exploris 120 mass spectrometer (Thermo Fisher Scientific, USA) equipped with an ESI ion source. The instrument was operated in Full MS-ddMS2 mode for concurrent MS1 and data-dependent MS/MS acquisition. Key source parameters were optimized as follows: sheath and auxiliary gas pressures were set at 40 and 10 (arbitrary units), respectively; the capillary temperature was maintained at 325 °C; and spray voltages were applied at +3.50 kV and −2.50 kV for positive and negative modes. Full MS scans (MS1) covered a mass range of m/z 100–1000 at a resolution of 60,000 FWHM. For MS/MS, the top 4 most intense ions per cycle were fragmented with a normalized collision energy of 30% and analyzed at a resolution of 15,000 FWHM, with dynamic exclusion enabled [26].

#### 2.14.4. Data Processing and Analysis

Raw data were converted into mzXML format using ProteoWizard and peak alignment, retention time correction, and peak area extraction were performed using the XCMS program, with a retention time tolerance of 0.5 min for consistent peak alignment across samples. To ensure data robustness, a rigorous quality control (QC) procedure was implemented using a pooled QC sample injected throughout the analytical sequence. After peak extraction, metabolic features with a relative standard deviation (RSD) > 30% in the QC samples were filtered out and excluded from subsequent analysis. Additionally, ion peaks with missing values greater than 50% within any experimental group were removed. Metabolites were annotated by matching exact mass (<25 ppm) and MS/MS fragments against a self-built database. Post-XCMS processing, peaks with >50% missing values per group were filtered. The filtered data were Pareto-scaled and subjected to multivariate analysis (PCA, PLS-DA, OPLS-DA) in SIMCA-P 14.1 and univariate analysis (*t*-test, fold change) in R. Significant differential metabolites were identified by combining a VIP score >1.0 from the OPLS-DA model with a univariate *p*-value < 0.05.

### 2.15. Statistical Analysis

Before statistical analysis, all data were tested for normality and homogeneity of variance. Statistical analysis and graphing were performed using GraphPad Prism 8.0. Significant differences between groups were analyzed using one-way or two-way analysis of variance (ANOVA), depending on the experimental design. For one-way ANOVA, and when a significant interaction or main effect was found in two-way ANOVA, post hoc comparisons were conducted using Tukey’s honest significant difference (HSD) test.

## 3. Results

### 3.1. Effects of Lycopene and SKQ1 on Sperm

#### 3.1.1. Lycopene and SKQ1 Improve Sperm Motility

**Figure 1:**
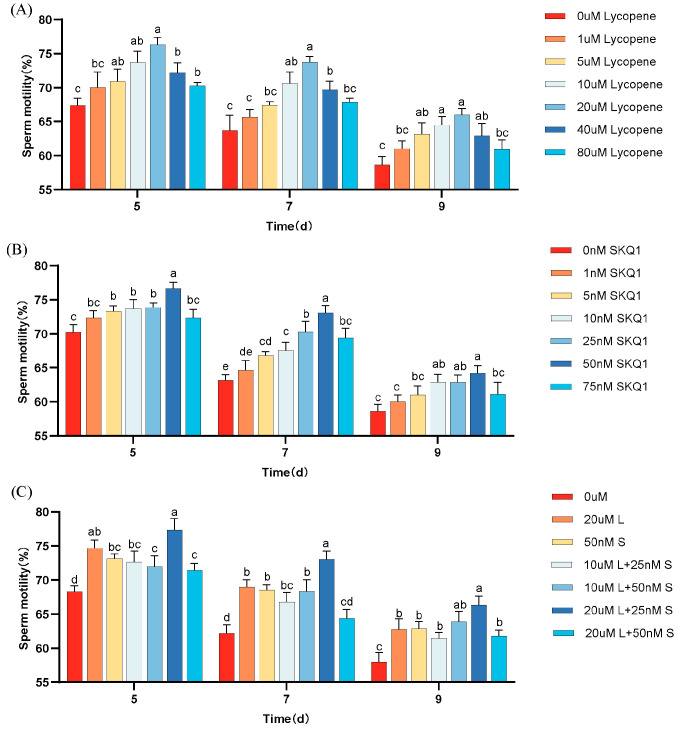
Effect of lycopene and SKQ1 on Sperm Motility Parameters. (**A**) lycopene alone treatment; (**B**) SKQ1 alone treatment; (**C**) Combined treatment of lycopene and SKQ1. Columns with different lowercase letters represent significant differences (*p* < 0.05). Values are expressed as mean ± standard deviation (n = 4).

The effects of different concentrations of lycopene and SKQ1 on sperm motility during 17 °C storage are shown in Figure 1. The results indicate that, compared to the control group, the addition of 20 μM lycopene and 50 nM SKQ1 significantly improved sperm motility during 17 °C storage (*p* < 0.05). The combination treatment of 20 μM lycopene and 25 nM SKQ1 exhibited the best effect (*p* < 0.05), although the effect decreased over time.

#### 3.1.2. Lycopene and SKQ1 Improve Sperm Acrosome and Membrane Integrity

**Figure 2:**
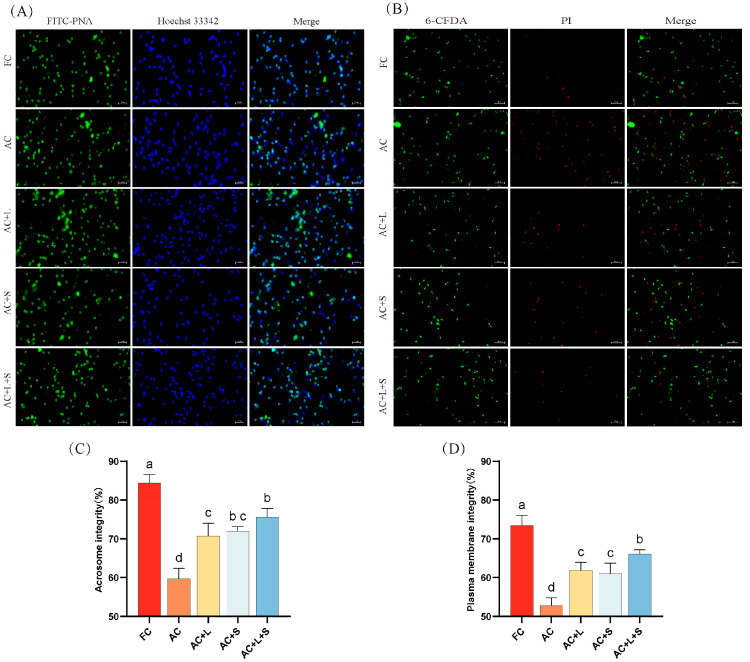
Effects of lycopene and SKQ1 on Acrosome Integrity and Membrane Integrity. (**A**) Fluorescence images of sperm acrosome integrity. Scale bar = 20 μm; (**B**) Fluorescence images of sperm membrane integrity. Scale bar = 50 μm; (**C**) Statistical analysis of sperm acrosome integrity. (**D**) Statistical analysis of sperm membrane integrity; FC (Fresh Control): Fresh sperm; AC (Aged Control): Sperm stored for seven days at 17 °C; AC + L (Aged Control + lycopene): Sperm with 20 μM lycopene; AC + S (Aged Control + SKQ1): Sperm with 50 nM SKQ1; AC + L + S (Aged Control + lycopene + SKQ1): Sperm with combined 20 μM lycopene and 25 nM SKQ1. Columns with different lowercase letters indicate significant differences (*p* < 0.05), values are presented as mean ± standard deviation (n = 5).

The effect of different concentrations of lycopene and SKQ1 on the acrosome integrity and membrane integrity of boar sperm during storage at 17 °C is shown in Figure 2. The results indicate that, compared to fresh sperm, both acrosome integrity and membrane integrity significantly decreased on the seventh day of storage (*p* < 0.05). The addition of 20 μM lycopene and 50 nM SKQ1 individually improved both acrosome and membrane integrity (*p* < 0.05). Moreover, the combined treatment of 20 μM lycopene and 25 nM SKQ1 slightly enhanced the effects.

#### 3.1.3. Lycopene and SKQ1 Reduce Oxidative Stress in Sperm

**Figure 3:**
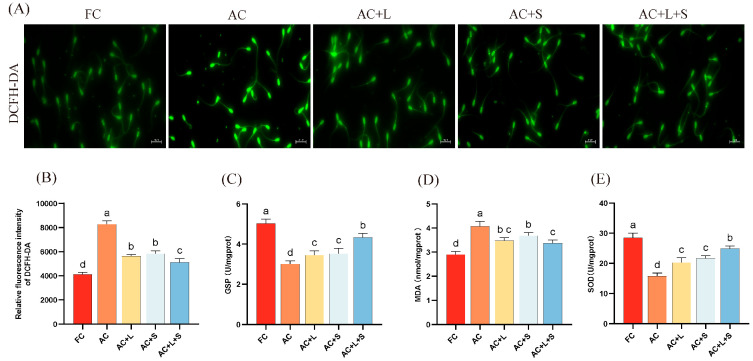
Effects of lycopene and SKQ1 on Sperm Oxidative Stress Levels. (**A**) Fluorescence results of sperm ROS content. Scale bar = 20 μm. (**B**) Statistical analysis of sperm ROS content. (**C**) Statistical analysis of sperm GSP activity. (**D**) Statistical analysis of sperm SOD activity. (**E**) Statistical analysis of sperm MDA content. Columns with different lowercase letters indicate significant differences (*p* < 0.05). Values are expressed as mean ± standard deviation (n = 5). FC (Fresh Control): Fresh sperm; AC (Aged Control): Sperm stored for seven days at 17 °C; AC + L (Aged Control + lycopene): Sperm with 20 μM lycopene; AC + S (Aged Control + SKQ1): Sperm with 50 nM SKQ1; AC + L + S (Aged Control + lycopene + SKQ1): Sperm with combined 20 μM lycopene and 25 nM SKQ1. Columns with different lowercase letters indicate significant differences (*p* < 0.05), values are presented as mean ± standard deviation (n = 5).

Furthermore, we assessed the effects of lycopene and SKQ1 on the ROS content and related antioxidant markers in boar sperm during 17 °C storage, as shown in Figure 3. From panel A, it is evident that after 7 days of storage, ROS levels increased compared to fresh sperm, with higher fluorescence intensity. However, upon addition of the two antioxidants and combined treatment, fluorescence intensity decreased, indicating lower ROS content than the control group. The results demonstrate that supplementation with 20 μM lycopene and 25 nM SKQ1 significantly reduced ROS and MDA levels in boar semen stored at 17 °C (*p* < 0.05), while increasing GSP and SOD enzyme activity (*p* < 0.05), thereby lowering oxidative stress levels and improving sperm quality.

#### 3.1.4. Lycopene and SKQ1 Improve Sperm MMP and ATP Levels

**Figure 4:**
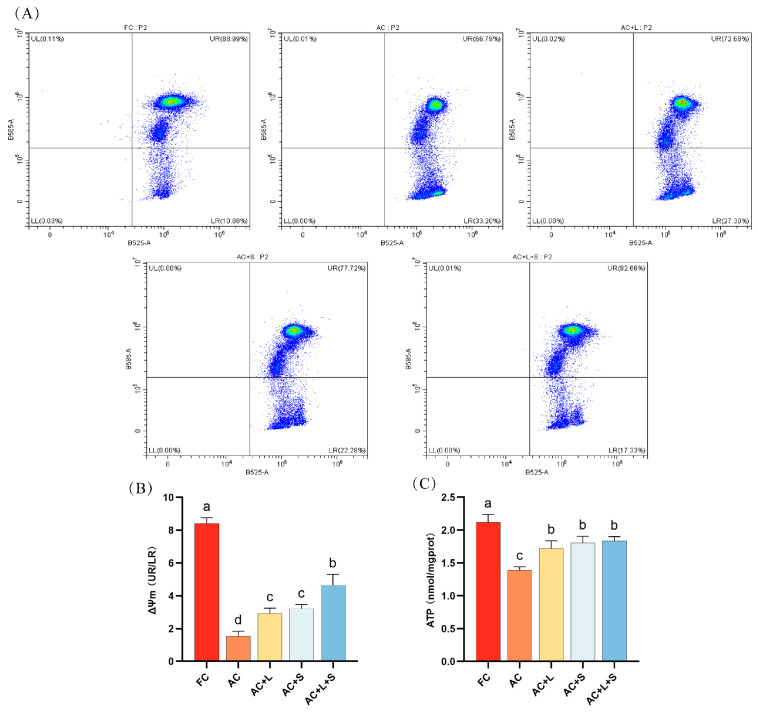
Effects of lycopene and SKQ1 on Sperm MMP and ATP Levels. (**A**) Flow cytometry analysis of sperm MMP. (**B**) Statistical analysis of sperm MMP levels. (**C**) Statistical analysis of sperm ATP content. Columns with different lowercase letters indicate significant differences (*p* < 0.05). Values are expressed as mean ± standard deviation (n = 5). FC (Fresh Control): Fresh sperm; AC (Aged Control): Sperm stored for seven days at 17 °C; AC + L (Aged Control + lycopene): Sperm with 20 μM lycopene; AC + S (Aged Control + SKQ1): Sperm with 50 nM SKQ1; AC + L + S (Aged Control + lycopene + SKQ1): Sperm with combined 20 μM lycopene and 25 nM SKQ1. Columns with different lowercase letters indicate significant differences (*p* < 0.05), values are presented as mean ± standard deviation (n = 5).

We also assessed the sperm MMP and ATP levels during 17 °C storage, as shown in Figure 4. Compared to fresh sperm, sperm stored for seven days exhibited a decrease in MMP levels, with the proportion of sperm exhibiting low MMP increasing to 33.20%. The addition of lycopene and SKQ1 mitigated this effect, with the best results observed in the group treated with 20 μM lycopene and 25 nM SKQ1, significantly increasing both sperm MMP and ATP levels (*p* < 0.05).

### 3.2. Sequencing Results and Analysis

**Figure 5:**
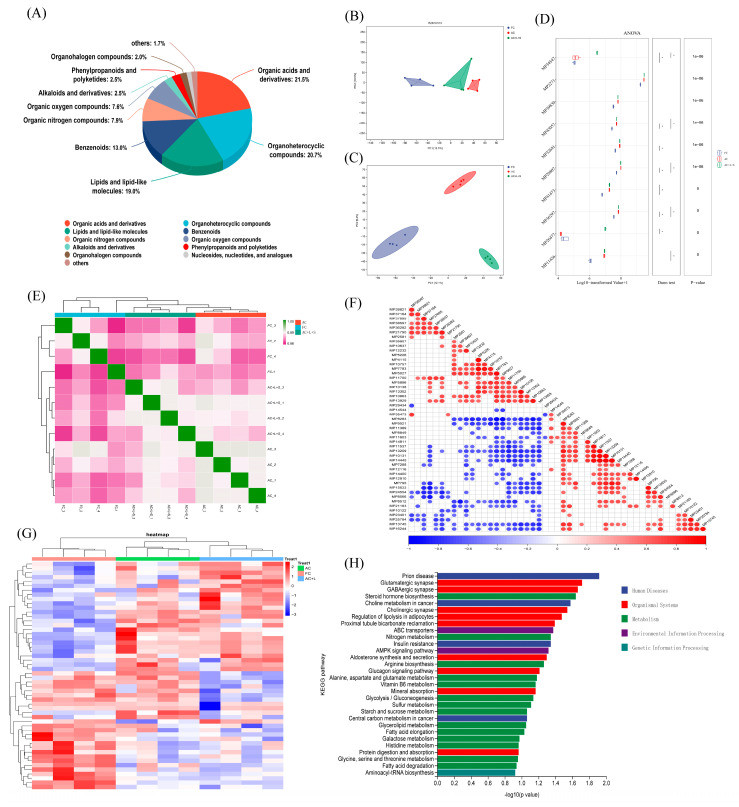
Untargeted Metabolomics Sequencing Results and Analysis of Sperm. (**A**) Metabolite identification analysis results. (**B**) Multivariate statistical PCA analysis results. (**C**) Multivariate statistical PLS-DA analysis results. (**D**) Multiple comparison analysis results. (**E**) Sample correlation analysis results. (**F**) Differential metabolite correlation analysis results. (**G**) Differential metabolite clustering analysis results. (**H**) Differential metabolite KEGG enrichment analysis results. FC (Fresh Control): Fresh sperm; AC (Aged Control): Sperm stored for seven days at 17 °C; AC + L + S (Aged Control + lycopene + SKQ1): Sperm with combined 20 μM lycopene and 25 nM SKQ1. Columns with different lowercase letters indicate significant differences (*p* < 0.05), values are presented as mean ± standard deviation (n = 4).

We performed untargeted metabolomics sequencing on four samples each from the FC, AC, and AC + L + S groups. Among the detected metabolites, 21.5% were classified as organic acids and derivatives, 20.7% as organoheterocyclic compounds, 19.0% as lipids and lipid-like molecules, and 13.0% as benzenoids, as shown in Figure 5A. The PCA results in Figure 5B reveal no significant overlap between the three sperm sample groups, with clear separation observed. Using a selection criterion of *p* < 0.05 for differential metabolites, we identified 52 differential metabolites across the three boar sperm groups, and the clustering heatmap is shown in Figure 5G. Compared to fresh sperm, 15 metabolites, including corticosterone, ceramide, and L-acety-L-carnitine, were elevated in the control group, whereas these metabolites decreased in the groups treated with 20 μM lycopene and 25 nM SKQ1. Conversely, 18 metabolites, such as 2-Tiglyl-L-carnitine and 3-Hydroxybutyryl-L-carnitine, were reduced in the control group but increased in the treatment groups. A KEGG pathway enrichment analysis of the 33 different metabolites exhibiting regular changes revealed that these metabolites were primarily enriched in 12 signaling pathways, including steroid hormone biosynthesis, ATP-binding cassette (ABC) transporter proteins, and AMP-activated protein kinase (AMPK) activation pathways (*p* < 0.05).

### 3.3. Western Blot Analysis Results

**Figure 6:**
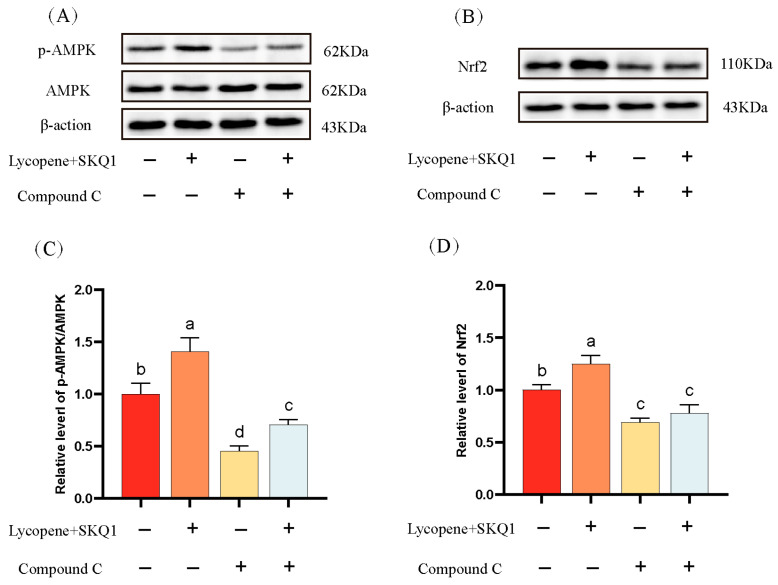
Western blot analysis of sperm protein expression. (**A**) Western blot results of sperm AMPK phosphorylation. (**B**) Western blot results of sperm Nrf2 expression. (**C**) Statistical analysis of sperm AMPK phosphorylation levels. (**D**) Statistical analysis of sperm Nrf2 expression levels. Columns with different lowercase letters indicate significant differences (*p* < 0.05). Data are presented as the mean ± standard deviation (n = 3).

AMPK is a critical energy sensor in sperm cells. It is activated under conditions of energy deficit to maintain cellular energy balance [27]. Nuclear factor erythroid 2-related factor 2 (Nrf2) is a key transcription factor involved in cellular responses to oxidative stress. Under oxidative stress, Nrf2 dissociates from its inhibitor Keap1, translocates to the nucleus, and binds to the antioxidant response elements (ARE), mitigating oxidative stress [28]. It has been reported that AMPK can promote the nuclear accumulation of Nrf2 via phosphorylation, leading to the upregulation of antioxidant enzyme expression [29]. In this study, we performed Western blotting to assess the p-AMPK and the expression of Nrf2 in sperm cells. As shown in Figure 6, the p-AMPK and the expression of Nrf2 were significantly elevated in the 20 μM lycopene and 25 nM SKQ1 treatment groups (*p* < 0.05). Upon the addition of 10 μM AMPK pathway inhibitor Compound C, both p-AMPK and Nrf2 expression were significantly reduced (*p* < 0.05). These results suggest that lycopene and SKQ1 may act through the AMPK/Nrf2 pathway to enhance sperm cell preservation quality.

## 4. Discussion

Compared to freezing, semen storage at 17 °C is simpler, more convenient, and widely used in practical production. However, during storage at 17 °C, sperm mitochondria continuously generate ROS. Sperm often lack sufficient endogenous antioxidants to eliminate ROS [30], and excessive ROS can lead to oxidative stress, which damages sperm vitality and quality. Therefore, the addition of exogenous antioxidants is required to maintain ROS balance and improve semen preservation efficiency. Lycopene is a natural antioxidant, has been shown to improve the in vitro preservation of semen in multiple species [31]. SKQ1, a novel mitochondrial-targeted antioxidant, has been studied less extensively. To date, no studies have investigated the effects of these two substances on boar sperm during 17 °C storage. Thus, our aim was to assess their effects on boar sperm quality during 17 °C storage, determine their optimal concentrations, and explore potential synergistic effects and underlying mechanisms.

Sperm vitality is a key indicator of sperm quality, directly influencing the success of sperm transport to the fertilization site during artificial insemination [20]. Our experimental results demonstrate that the addition of 20 μM lycopene and 50 nM SKQ1 effectively improves sperm vitality and energy levels. The combination treatment of 20 μM lycopene and 25 nM SKQ1 further enhances this effect, with sperm vitality exceeding 70% on day 7 of storage at 17 °C, meeting the requirements for practical production applications. Fluorescence staining also revealed that on day 7 of storage, some sperm in the control group showed acrosomal loss and damage, with a significant reduction in mitochondrial membrane potential (MMP). This may be due to the lack of exogenous antioxidant protection, resulting in accumulated excess ROS that damage polyunsaturated fatty acids in sperm, producing large amounts of malondialdehyde (MDA), leading to mitochondrial electron transport dysfunction and a decline in MMP [32,33]. In the lycopene and SKQ1-treated groups, severe acrosomal loss was almost absent, and the number of sperm with acrosomal damage was reduced compared to the control group. The integrity of both the acrosome and plasma membrane was also significantly improved.

Interestingly, we observed that the group treated with 20 μM lycopene alone exhibited better antioxidant capacity, with lower ROS and MDA levels compared to the 50 nM SKQ1 treatment group. On the other hand, the group treated with 50 nM SKQ1 alone showed higher MMP and ATP levels, indicating a higher energy metabolism level than the lycopene treatment group. Compared to individual treatments, the combination of lycopene and SKQ1 provided enhanced protective effects, likely due to their complementary mechanisms. lycopene, as a natural antioxidant, mainly acts by scavenging intracellular ROS [34], while SKQ1, a mitochondrial-targeted antioxidant, prevents mitochondrial dysfunction and improves sperm energy metabolism [35,36]. Together, they offer dual protection against oxidative stress and mitochondrial dysfunction. Previous studies on the combined use of antioxidants have supported this approach. For instance, Zhao et al. [37] demonstrated that adding 1400 IU/mL Vitamin C (Vc) and 0.12 IU/mL Vitamin E (Ve) protected bull sperm quality during freezing and thawing, while Pei et al. [38] showed that a combination of 0.1 mM Apigenin and 0.15 mM Ferulic acid maximized the quality of boar sperm after freezing and thawing. These synergistic effects highlight the importance of combined antioxidant treatments in improving sperm preservation.

Subsequently, we conducted metabolomic analysis of boar sperm. Using *p* < 0.05 as the threshold for screening differential metabolites, a total of 52 differential metabolites were identified across the three sperm groups. The objective of the antioxidant supplementation experiment was to make the physiological and biochemical state of sperm after a period of preservation more closely resemble that of fresh sperm. Therefore, we performed a secondary screening of the 52 differential metabolites and selected 33 that exhibited consistent and directional changes. Among these, L-Acetylcarnitine, a key molecule in energy metabolism, transports acetyl groups into the mitochondrial tricarboxylic acid cycle for oxidative energy production. It is crucial for sperm motility and maturation, and its levels are typically associated with asthenozoospermia and oxidative stress [39,40]; 2-Tiglylcarnitine is an intermediate in leucine metabolism and fatty acid oxidation pathways, and its aberrant levels are often linked to mitochondrial dysfunction [41]; (R)-3-Hydroxybutyrylcarnitine is an intermediate in ketone body metabolism and fatty acid β-oxidation; its precursor, (R)-3-Hydroxybutyric acid, is an important energy substrate. Previous studies have found that it can help improve myocardial energy metabolism and support mitochondrial function, thereby reducing oxidative stress [42]. Additionally, bioactive lipids such as Myristamide and (R)-palmitic monoisopropanolamide were identified, which play significant roles in cellular membrane structure [43,44].

KEGG enrichment analysis of these differential metabolites revealed significant enrichment (*p* < 0.05) in 12 key pathways, including steroid hormone biosynthesis, regulation of lipolysis in adipocytes, ABC transporters, choline metabolism, and the AMPK signaling pathway. Notably, energy pathways such as glycolysis and gluconeogenesis were not significantly enriched (*p* > 0.05). These results indicate that the supplementation of lycopene and SKQ1 during semen preservation likely exerts their effects by influencing sperm energy metabolism processes and mitochondrial function. This intervention appears to improve the integrity of the sperm acrosome and plasma membrane, ameliorate cellular stress, and enhance the microenvironment, thereby ultimately improving sperm quality.

AMPK is essentially a heterotrimeric complex composed of α, β, and γ subunits. It is regarded as a cellular energy sensor and is a key signaling kinase in sperm cells. Once activated, AMPK promotes ATP production through the glycolysis and gluconeogenesis signaling pathways, enhances mitochondrial membrane potential, and conserves ATP by shutting down biosynthetic pathways [45]. Current research suggests that AMPK is positively correlated with sperm vitality, playing a crucial role in regulating sperm motility, mitochondrial activity, and maintaining the normal physiological functions of sperm [27,46]. Recent studies have shown that activated AMPK can phosphorylate specific amino acid sites on the Nrf2 protein [29], facilitating the nuclear translocation of Nrf2 to bind with antioxidant response elements (ARE), thereby initiating the transcription of downstream genes such as SOD, GSP, and ABCC1. SOD [47] and GSP [48] are important antioxidant enzymes in male reproductive cells, capable of scavenging excessive ROS produced by mitochondria, preventing oxidative stress [49].Our study found that the addition of 20 μM lycopene and 25 nM SKQ1 significantly increased the activity of SOD and GSP in sperm cells, while reducing the levels of ROS and MDA in sperm cells. Combined with the sperm metabolomics analysis results, we hypothesize that the joint treatment with lycopene and SKQ1 may exert its antioxidant effects via the AMPK/Nrf2 pathway and improve sperm cell mitochondrial membrane potential (MMP) and ATP levels through glycolysis.

**Figure 7:**
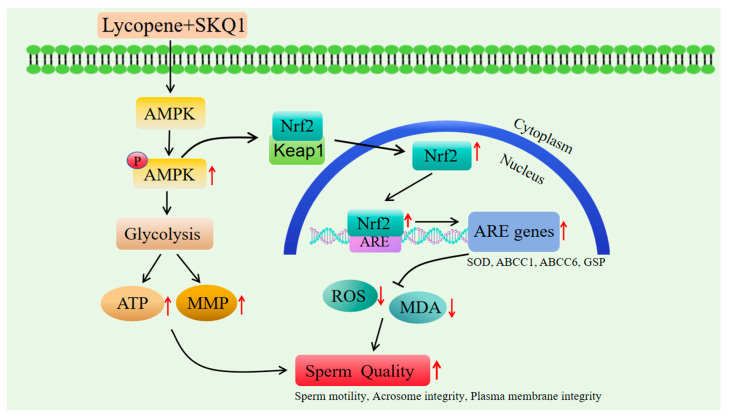
The specific mechanism of lycopene and SKQ1 improve boar sperm quality during storage at 17 °C.

To verify this hypothesis, we performed subsequent Western blot experiments. The results showed that after adding 20 μM lycopene and 25 nM SKQ1, the p-AMPK and Nrf2 expression in sperm cells significantly increased. However, when we added 10 μM Compound C (an AMPK inhibitor), both p-AMPK and Nrf2 expression levels significantly decreased. Therefore, we conclude that the combined treatment with lycopene and SKQ1 activates the AMPK signaling pathway, leading to the activation of AMPK in the cytoplasm, which subsequently triggers Nrf2 to translocate into the nucleus and bind with ARE. This process enhances the antioxidant enzyme activity and energy levels in sperm cells, thereby improving the quality of boar sperm during storage at 17 °C. The specific mechanism of action is summarized in Figure 7.

Previous studies have demonstrated the beneficial effects of antioxidants such as Rosmarinic acid [50], Resveratrol [51] and Astaxanthin [8] on boar sperm preservation. However, these antioxidants typically act in a non-specific manner, targeting general oxidative stress without considering sperm cell energy metabolism or the underlying mechanisms. In contrast, lycopene and SKQ1 provide a more specific mechanism by targeting the AMPK/Nrf2 pathway, thereby enhancing antioxidant defense and mitochondrial function in sperm cells. The findings of this study hold significant implications for improving sperm preservation and artificial insemination efficiency in commercial breeding operations. By improving sperm vitality and extending storage time, the combined use of lycopene and SKQ1 can enhance the success rate of artificial insemination, increase the utilization efficiency of superior boars, and potentially extend the reach of semen distribution networks. From a practical standpoint, this formulation is highly attractive: lycopene, as a naturally sourced antioxidant, offers a cost-effective solution, while SKQ1, a synthetic mitochondrial-targeted antioxidant, is highly effective at nanomolar concentrations, ensuring a low final cost per dose. This method may also be applied to sperm preservation in other livestock species, offering a practical and economically viable strategy to improve overall reproductive performance.

## 5. Conclusions

In summary, the combined use of lycopene and SKQ1 is a promising and cost-effective strategy that improves boar sperm quality during 17 °C storage. Their ability to mitigate oxidative stress and enhance energy metabolism optimizes sperm preservation techniques. These findings not only deepen our understanding of antioxidant mechanisms in sperm but also provide a practical and reliable solution for increasing artificial insemination success rates in commercial livestock production.

## Figures and Tables

**Table 1:** Antibody basic information.

Antibody Name	Product Number	Company
Phospho-AMPKalpha	50081S	CST (Danvers, MA, USA)
AMPKalpha	5831T	CST (Danvers, MA, USA)
Nrf2	EP1808Y	Abcam (Cambridge, UK)
Anti-beta Actin	GB113225-100	Servicebio (Wuhan, China)

## Data Availability

The data presented in this study are available on request from the corresponding author.
